# A familial case of Birt–Hogg–Dubé syndrome complicated with lung cancer: a case report and literature review

**DOI:** 10.3389/fmed.2025.1581786

**Published:** 2025-06-24

**Authors:** Jingjing Feng, Yunxia Yu, Yi Liu, Yi Ding, Junyi Mu, Rong Jiang, Lokesh Sharma, Zhijun Jie

**Affiliations:** ^1^Center of Community-Based Health Research, Department of Pulmonary and Critical Care Medicine, Shanghai Fifth People’s Hospital, Fudan University, Shanghai, China; ^2^Shanghai Public Health Clinical Center, Shanghai Emerging and Re-Emerging Institute, Fudan University, Shanghai, China; ^3^Division of Pulmonary, Allergy, Critical Care, and Sleep Medicine, Department of Medicine, School of Medicine, University of Pittsburgh, Pittsburgh, PA, United States

**Keywords:** BHD, lung cancer, FLCN, gene mutation, familial case

## Abstract

The Birt–Hogg–Dubé (BHD) syndrome is a rare, autosomal dominant disorder caused by germline mutations in the FLCN gene, which encodes a tumor suppressor protein. The syndrome is characterized by cutaneous fibrofolliculomas, pulmonary cysts, spontaneous pneumothoraces, and renal tumors. We report a rare familial case of BHD complicated by primary lung adenocarcinoma in a 69-year-old man. The patient presented with a persistent cough, bilateral pulmonary cysts, and a 4 × 3 cm tumor in the left lung lobe. Genetic testing revealed a novel pathogenic FLCN mutation (c.295_311del p.Asp99Ter) in the patient and several family members, including his son and brother, both of whom exhibited pulmonary cysts and histories of pneumothoraces. The patient was diagnosed with invasive lung adenocarcinoma. Although tumor progression was stabilized with treatment, the clinical course was complicated by severe hyperthyroidism, liver injury, and myelosuppression. This case highlights the complexities of managing lung cancer in BHD patients and suggests a potential role for FLCN mutations in tumorigenesis. Our findings underscore the importance of early diagnosis, genetic testing, and a multidisciplinary approach to the management of BHD in order to improve patient outcomes and prevent complications. In addition, we conducted a literature review of previously reported FLCN mutations in BHD syndrome.

## Introduction

The Birt–Hogg–Dubé (BHD) syndrome is a rare inherited autosomal dominant disease caused by germline mutations in the folliculin (FLCN) gene (1). The typical clinical presentation includes cutaneous fibrofolliculomas, multiple pulmonary cysts, recurrent spontaneous pneumothoraces, and renal tumors (2). The FLCN gene was identified as a tumor suppressor gene, located on chromosome 17p11.2 (3). So far, over 800 pathogenic variants and more than 1,200 individuals with BHD have been reported worldwide in numerous affected families (4). Most of the mutations are protein-truncating, including frameshift, nonsense, or splice-site variants, which lead to a loss of function of folliculin (5). We herein present a novel FLCN mutation-mediated case of BHD complicated by primary lung cancer and review the pertinent literature. BHD is a rare syndrome, with limited knowledge among physicians. Many families with BHD may remain undiagnosed, given its rare prevalence and limited exposure of physicians to this disease. It is crucial to improve diagnosis and its management. Here, we present a case of BHD, and we hope to draw the attention of physicians to this rare inherited disorder.

## Case report

### Clinical presentation

**Figure 1:**
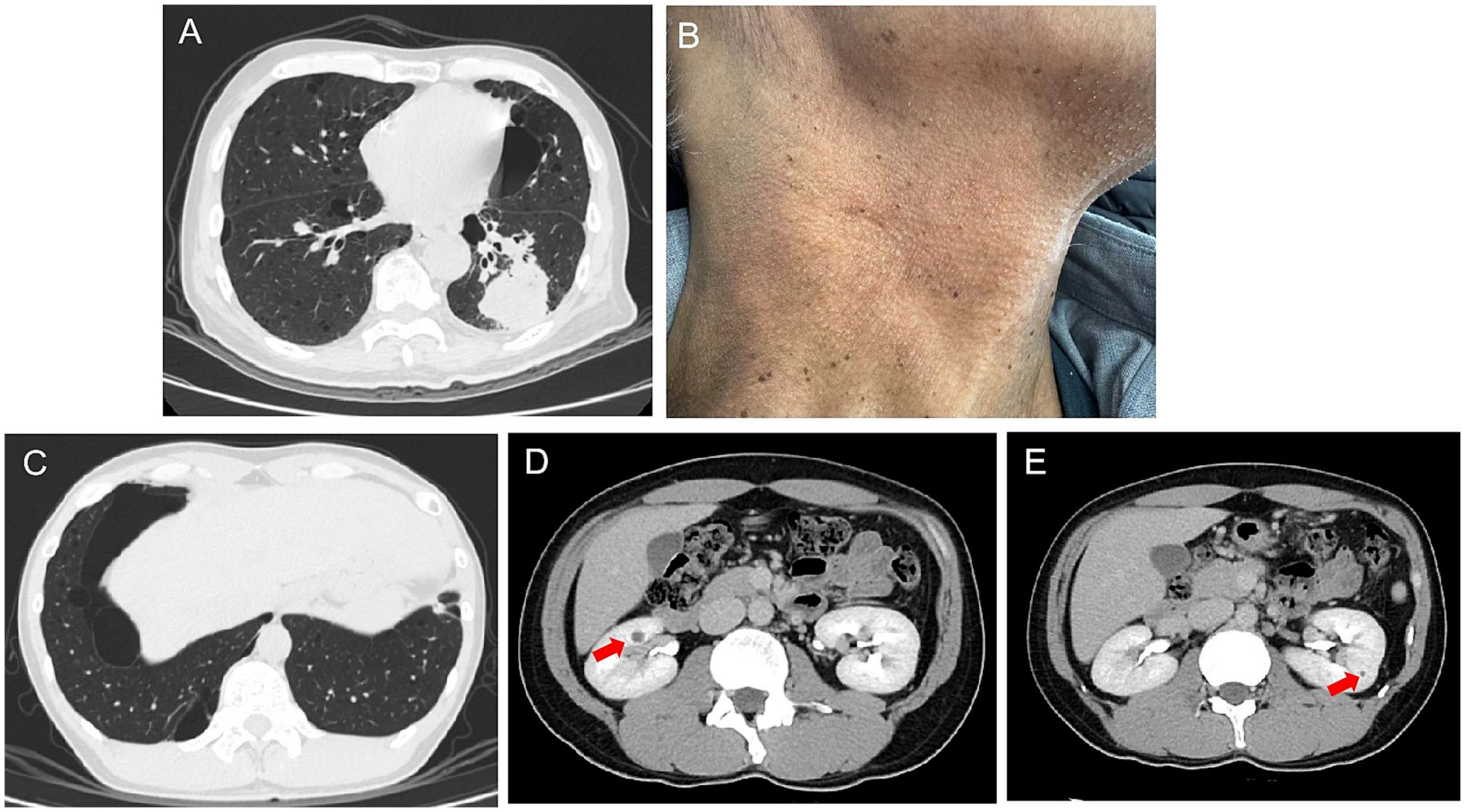
**(A)** Chest computed tomography (CT) scan of the patient. **(B)** Cutaneous fibrofolliculomas observed on this patient’s neck. **(C)** Chest CT scans of his son reveal multiple thin-walled parenchymal cysts. **(D,E)** Enhanced abdominal CT of his son indicates tumors in both of his kidneys, indicated by a red arrow.

**Figure 2:**
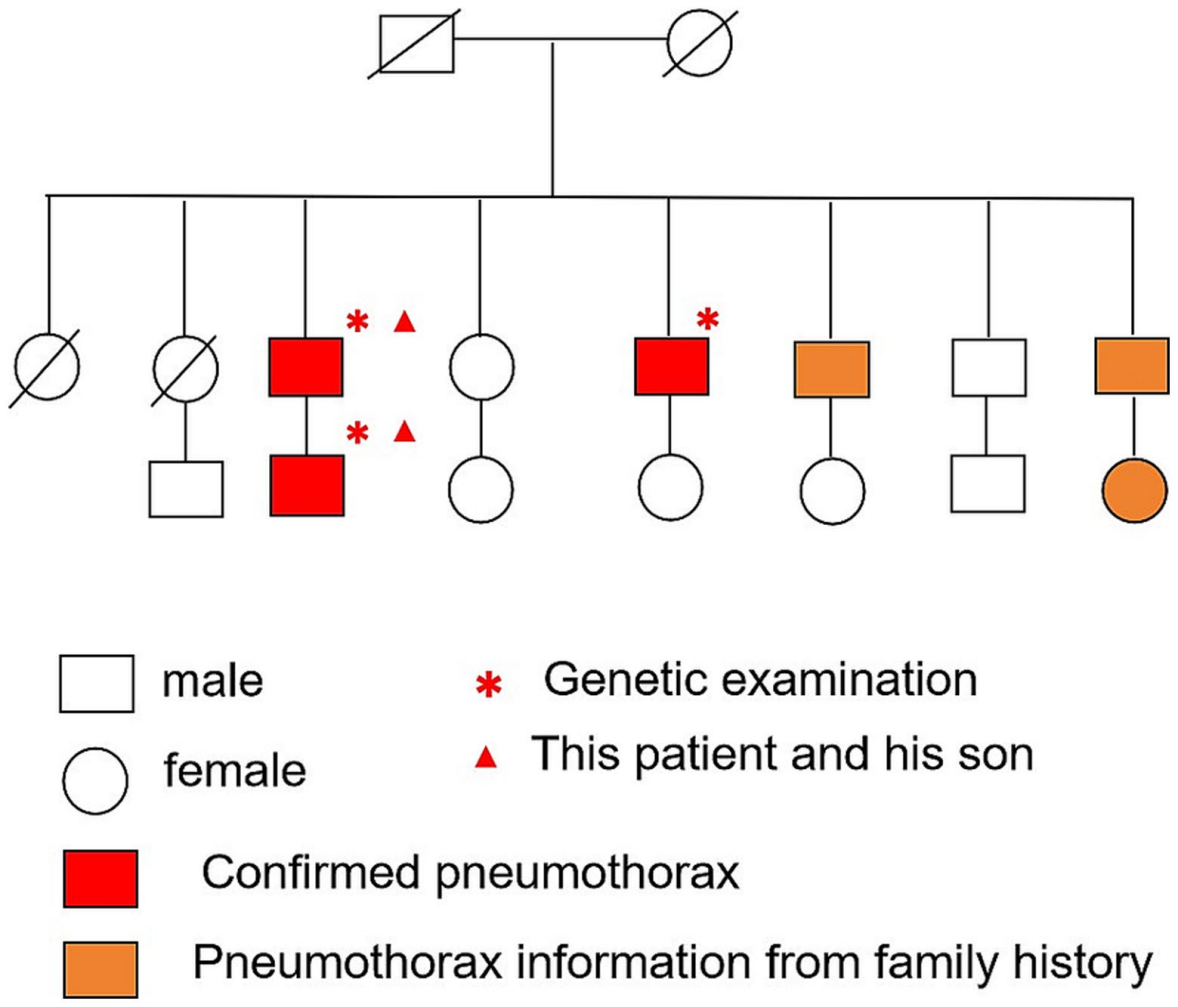
Pedigree chart of the patient’s family in three generations.

A 69-year-old man was admitted to Zhongshan Hospital, Fudan University, Shanghai, due to a cough lasting for months. A computed tomography scan demonstrated bilateral multiple cysts and a size 4 × 3 cm tumor in his left lung lobe in November 2023 (Figure 1A). Positron emission tomography-computed tomography showed metastases in the left hilar and mediastinal lymph nodes, right lobe of the liver, bilateral adrenal glands, and multiple bones. He was diagnosed with pulmonary adenocarcinoma via bronchoscopy-guided transbronchial needle aspiration biopsy (EBUS-TBNA), with stage T3N2M1c IVB (Eighth Edition TNM Cancer Staging System). A genetic test of the tumor was conducted. A Kirsten Rat Sarcoma Viral Oncogene Homolog (KRAS) exon 2 c.38G>A (p.G13D) mutation was detected in the tumor tissue, whereas mutations in the epidermal growth factor receptor (EGFR) and anaplastic lymphoma kinase (ALK) were negative. Therefore, no suitable mutations relevant to targeted treatment options were identified. For further treatment, the patient was transferred to our hospital. Physical examination showed small papules ranging from 0.5 to several millimeters in diameter were noticed on this patient’s neck and face (Figure 1B). The patient has been a smoker for 30 years but quit smoking a year ago. He had no prior history of exposure to any other known toxic materials. The patient had previously experienced one episode of left-sided spontaneous pneumothorax, which improved with conservative treatment. His medical and family history revealed that, besides him, three of his brothers and his son had histories of pneumothorax. Upon receiving informed consent, his son’s medical history was reviewed based on his annual health checkup. His son’s chest CT scan indicated the presence of pulmonary cysts as well (Figure 1C). Furthermore, an enhanced abdominal MRI scan of his son revealed tumors in both kidneys (Figures 1D,E). Other immediate family members, including his two older brothers, have also suffered from spontaneous pneumothorax. The family tree is shown in Figure 2.

**Figure 3:**
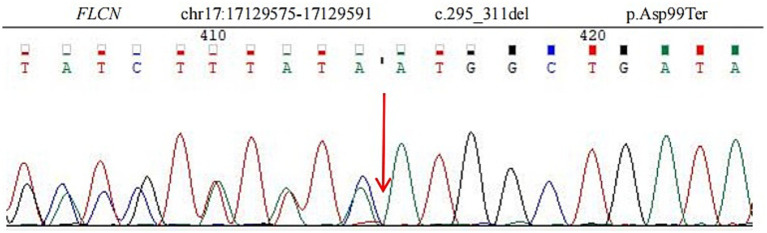
Sequence analysis showed a deletion mutation (c.295_311del p.Asp99Ter) in exon 5 of the FLCN gene. The deletion of nucleotides 295 to 311 in the coding region leads to the mutation of amino acid 99 from aspartic acid to termination.

**Figure 4:**
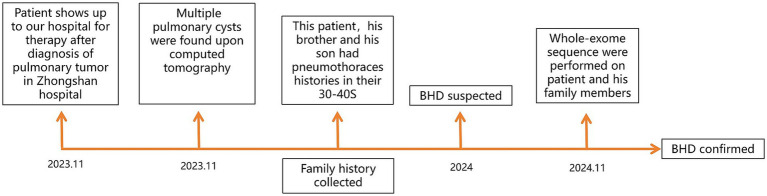
Diagnostic process timeline.

Given the typical lung tomography findings and the history of pneumothoraces, including those in other family members, a familial case of BHD was suspected. We performed whole-exome sequencing on the patient, his brother, and his son to detect the presence of any mutations related to BHD. The coding region of the FLCN gene, consisting of exons 4 to 14, was amplified with polymerase chain reaction (PCR) using oligonucleotide primers and was sequenced by Sanger sequencing. The FLCN mutation (c.295-311del p.Asp99Ter) in exon 5 was detected in all three of them (Figure 3), but we did not find any other mutations. This allowed us to make a positive diagnosis of BHD in the patient and his family (see Figure 4).

Following confirmation of lung cancer in January 2024, he was treated with pemetrexed and bevacizumab from January to February 2024. The treatment of the patient for his malignancy was complicated by unexpected side effects. He experienced severe hyperthyroidism after two doses of treatment and developed severe liver injury during treatment for hyperthyroidism. His alanine aminotransferase (ALT) increased to 261 U/L (normal range <41 U/L), and total bilirubin increased to 50.9 μmol/L (normal range <21 μmol/L). Upon recovery from liver injury and hyperthyroidism. Upon re-evaluation of the patient’s tumor staging, we observed tumor progression: the tumor continued to grow in the left lung, while new metastatic lesions were detected in the right lung. Based on these findings, his tumor control status was assessed as progressive disease (PD). Consequently, from March 2024 to February 2025, the patient received combination therapy with sintilimab, pemetrexed, and bevacizumab, administered every 3 weeks. This treatment regimen effectively stabilized the disease, and subsequent assessments indicated stable disease (SD). In December 2024, due to anemia secondary to myelosuppression, the dosage of pemetrexed was reduced by 20%, and one treatment cycle was withheld. Notably, no recurrences of hyperthyroidism and liver injury were observed during this period. However, in February 2025, the patient developed myelosuppression following treatments, which led to complications including pneumonia, anemia, and thrombocytopenia. Despite supportive care, his condition deteriorated, and he unfortunately passed away on 28 February 2025 (see Figure 4).

## Discussion

BHD syndrome is an autosomal dominant inherited disorder caused by germline mutations in the FLCN gene, located on chromosome 17p11.2, which encodes a highly conserved tumor suppressor protein. BHD was first described in 1977 by Canadian scientists Birt et al. (6). The classic clinical manifestations include lung cysts, characteristic skin lesions, and renal tumors. Lung cysts significantly increase the risk of spontaneous pneumothorax, whereas cutaneous manifestations—such as fibrofolliculomas, trichodiscomas, and acrochordons—primarily occur on the face, neck, and upper trunk (7). Our patient exhibited many of these features, raising clinical suspicion for BHD.

As a rare disorder, BHD is likely underdiagnosed and may be mistaken for other conditions that cause primary spontaneous pneumothorax, such as emphysema, lymphangioleiomyomatosis (LAM), pulmonary Langerhans cell histiocytosis (PLCH), or other cystic lung diseases (8). Early diagnosis is critical to enabling appropriate renal cancer screening for patients and affected family members. The most common clinical manifestation of BHD is spontaneous pneumothorax, particularly in individuals with a family history of pneumothorax. High-resolution computed tomography (HRCT) is strongly recommended to detect diffuse lung cysts. When BHD is suspected, a thorough personal and family history including skin lesions, pneumothoraces, lung cysts, and renal tumors should be obtained. A positive family history should prompt genetic testing to confirm the diagnosis.

A diagnosis of BHD requires that a patient meets either one major criterion or two minor criteria (9). Major criteria include: (a) at least five skin lesions (trichodiscomas or fibrofolliculomas), with at least one confirmed histopathologically and appearing in adulthood or (b) a known pathogenic mutation in the FLCN gene. Minor criteria include: (a) bilateral lung cysts predominantly at the lung base without another known cause (with or without spontaneous pneumothorax); (b) renal involvement, such as early-onset renal cancer (before age 50), multifocal or bilateral renal cell carcinoma, or chromophobe/oncocytic tumors; or (c) a family history of BHD in a first-degree relative. In our case, the patient fulfilled both major criteria—skin lesions and a confirmed FLCN gene mutation—as well as two minor criteria, including multiple bilateral pulmonary cysts and a history of spontaneous pneumothorax. These findings collectively confirmed the diagnosis of BHD.

BHD patients typically develop renal tumors, whereas only a few cases of lung cancer associated with BHD have been reported to date. In 2016, 14 pulmonary neoplastic lesions were identified in seven patients with BHD (10). In 2020, Goto et al. (11) reported a familial case of BHD complicated by various cancers and summarized four previous cases of lung cancer associated with BHD. A 2024 study from Eastern China summarized the clinical and genetic characteristics of 100 consecutive BHD patients and identified three cases of concomitant lung cancer, all of which were pathologically classified as adenocarcinoma (12). In our case, the patient has lung cancer with BHD syndrome, and a previously unreported FLCN mutation was detected at chr17:17129575–17129591 in exon 5 (c.295_311del p.Asp99Ter). This variant has not been documented in gnomAD exomes or genomes, making it a novel pathogenic mutation of the FLCN gene associated with BHD syndrome.

FLCN is expressed in multiple tissues, including the lungs, skin, and kidneys. Animal studies have shown that the FLCN gene mutations can spontaneously lead to renal cysts, adenomas, and carcinomas (13). The biological functions of FLCN are not yet fully understood, but it is reported to play roles in cell growth, proliferation, and survival, mainly through interactions with the mechanistic target of rapamycin (mTOR) signaling pathway (14–16). FLCN mutations may contribute to tumorigenesis in the lungs, and smoking could accelerate tumor development in BHD patients (17). Cigarette smoking has been shown to promote airway epithelial cell death through enhanced proteolysis, which induces FLCN loss (18). Our patient’s smoking history may have played a role in the development of lung adenocarcinoma. Furuya et al. (10) found that pulmonary neoplasms of peripheral adenocarcinomatous lineage in BHD patients frequently exhibit loss of heterozygosity (LOH) of FLCN along with mTOR pathway activation. However, it remains unclear whether synergism between EGFR/KRAS mutations and FLCN *LOH* accelerates tumor progression in the lung. Our patient has a *KRAS* mutation detected in the tumor tissue, but not in the blood sample. It is unclear whether the FLCN mutation contributes to the KRAS mutation in lung cancer.

To date, research has not established a definitive correlation between the type or location of FLCN variants and specific clinical phenotypes in BHD. However, some studies have suggested possible genotype–phenotype associations. For example, Toro et al. (19) reported that individuals with mutations in exon 9 had a higher number of pulmonary cysts, whereas mutations in exons 9 and 12 were linked to increased instances of pneumothorax. Another study found that carriers of the c.1300G>C (59%) or c.250-2A>G (77%) mutations had a significantly higher risk of pneumothorax compared to those with the hotspot mutation c.1285dup (37%) (20). Additionally, the c.1285dupC variant has been associated with a significantly increased risk of colorectal cancer compared to the c.610delGGinsTA variant (21). Although the comprehensive molecular mechanism is unclear, the dysregulation of the mTOR pathway may result in altered cell growth and protein synthesis, thereby leading to tumorigenesis. Recent studies have also indicated that FLCN suppresses transcription factor E3 and transforming growth factor (TGF)-β, which may additionally lead to the development of renal tumors in BHD patients (22, 23), highlighting the significance of these pathways in BHD-related tumorigenesis.

It is noteworthy that, in the context of lung cancer diagnosis and treatment for BHD syndrome patients, the presence of multiple pulmonary cysts and impaired lung function makes obtaining tissue samples through surgical or percutaneous biopsy particularly challenging.

During tumor treatment, this patient developed severe hyperthyroidism and myelosuppression. While hyperthyroidism is commonly associated with immunotherapy, it is important to note that the patient did not receive immunotherapy during the first 2 months of treatment. His liver injury was attributed to propylthiouracil, a standard medication for hyperthyroidism. The underlying cause of his thyroid dysfunction remained unclear, but anticancer medications were reintroduced without the recurrence of these side effects. Due to the progression of his lung tumor, sintilimab was added to his treatment regimen for better disease control. However, with continued treatment, he experienced persistent myelosuppression, likely related to prolonged pemetrexed use. Even after dose reduction, myelosuppression and its severe complications persisted.

This case exemplifies the heightened risk of complications in BHD patients undergoing anticancer therapy. Furthermore, due to the presence of multiple pulmonary cysts and impaired lung function, BHD patients may have unique challenges or potential risks when undergoing chemotherapy or immunotherapy for lung cancer. However, current evidence directly comparing the incidence of adverse events between BHD patients and the general population is lacking. Further studies are needed to clarify whether BHD confers an increased risk of treatment-related complications.

## Data Availability

The datasets presented in this study can be found in online repositories. The names of the repository/repositories and accession number(s) can be found in the article/supplementary material.
